# CEACAM6 is a prognostic biomarker and potential therapeutic target for gastric carcinoma

**DOI:** 10.18632/oncotarget.19415

**Published:** 2017-07-20

**Authors:** Guo-Qing Ru, Yong Han, Wei Wang, Yuan Chen, Hui-Ju Wang, Wen-Juan Xu, Jie Ma, Meihua Ye, Xi Chen, Xiang-Lei He, Balázs Győrffy, Zhong-Sheng Zhao, Dongsheng Huang

**Affiliations:** ^1^ Department of Pathology, Zhejiang Provincial People's Hospital, Hangzhou, Zhejiang, PR China; ^2^ Clinical Research Institute, Zhejiang Provincial People's Hospital, Hangzhou, Zhejiang, PR China; ^3^ VIP Medical Center, Zhejiang Provincial People's Hospital, Hangzhou, Zhejiang, PR China; ^4^ Momentum Cancer Biomarker Research Group, Research Centre for Natural Sciences, Hungarian Academy of Sciences, Budapest, Hungary; ^5^ Second Department of Pediatrics, Semmelweis University, Budapest, Hungary

**Keywords:** CEACAM6, gastric carcinoma, overall survival, metastasis, chemotherapeutics

## Abstract

This study aims to investigate the prognostic power of carcinoembryonic antigen-related cell adhesion molecule 6 (CEACAM6) in gastric cancer (GC) and its potential role in cancer development and progression. Data mining results show that CEACAM6 is overexpressed in gastric cancer and is correlated with lymph node metastasis. Subsequently, immunohistochemical staining was performed to determine CEACAM6 protein levels in paraffin gastric tumor specimens. Real-time reverse-transcription-polymerase chain reaction (RT-PCR) was conducted to detect CEACAM6 mRNA levels in fresh GC samples. CEACAM6 protein and mRNA levels were significantly up regulated in GC compared with paired normal mucosa. The IHC staining intensity of CEACAM6 was positively correlated with tumor size, Lauren's classification, vascular invasion, lymph node metastasis, distant metastasis, and TNM stage. CEACAM6 expression was inversely correlated with the five-year survival rate of GC patients. Cox multivariate analysis results demonstrated that the overall survival was independently correlated with CEACAM6 expression. A significant association was observed between CEACAM6 and distant metastases. Network analysis of downstream gene signatures revealed several hub genes such as SRC and DNM1L etc. which may mediating tumor promoting functions of CEACAM6. Further data mining discovered that Tamoxifen etc. could be therapeutic alternatives for gastric patients with CEACAM6 overexpression. Collectively, CEACAM6 overexpression is a common characteristic of GC and is associated with poor 5 year survival rate in GC. Besides, potential molecular mechanisms and treatment options were also provided.

## INTRODUCTION

Gastric cancer (GC) is one of the most common causes of cancer-related mortality worldwide with an estimated 951,600 new cases and 723,100 deaths occurred in 2012 [1, 2]. Similar to other malignancies, the development of GC is a multi-step process. Early detection and appropriate treatment remains promising approach for improving the long-term prognosis of patients with GC. Despite advances in the diagnosis and treatment of GC in the last decade, prognosis for patients with advanced GC remains poor [3, 4].

Various cell adhesion molecules (CAMs) participate in the invasion and metastasis process during cancer progression [5]. Dysregulation of CAM expression and function has been found in malignant transformation. Carcinoembryonic antigen-related cell adhesion molecule (CEACAM) family are transmembrane glycoproteins that belong to the immunoglobulin superfamily. They are involved in several biological processes [6], such as cell growth, differentiation, cell recognition, immune response, and adhesion [7–10]. Different members of the CEACAM family may have different functions [11, 12]. There are four members of CEACAMs (namely, CEACAM1, CEACAM5, CEACAM6 and CEACAM7) have been found in epithelia [13]. Theses CEACAMs members can mediate several cell signaling pathways and lead to various functions, such as tumor suppression/promotion, angiogenesis, lymphocyte activation, cell cycle and adhesion [14–16]. CEACAMs are often co-expressed in several tumor types. For instance, CEACAM 1, 5, and 6 are co-expressed in endometrial, lung, ovarian, cervical, breast and colon cancers [17–19].

CEACAM6, also known as *CD66c*, belongs to the carcinoembryonic antigen (CEA) family [20]. It is overexpressed in several cancer types (such as ovarian, colon, breast and non-small cell lung cancers) and could promote cancer progression by inducing epithelial-mesenchymal transition (EMT) [21–23]. In our previous study, we found CEACAM6 was up regulated (ratio ≥ 2) in gastric tumor tissues by performing the Affymetrix GeneChip HG-U133A2.0 array [24]. However, few studies have provided information regarding the expression patterns of CEACAM6 and their functions in GC.

In this study, we will examine the expression of CEACAM6 in GC and normal mucosa, the correlation between CEACAM6 expression and clinicopathological factors, the prognostic value of CEACAM6 in GC and potential molecular functions of CEACAM6 in GC progression.

## RESULTS

### CEACAM6 is overexpressed in gastric cancer and is correlated with lymph node metastasis in several datasets

**Figure 1:**
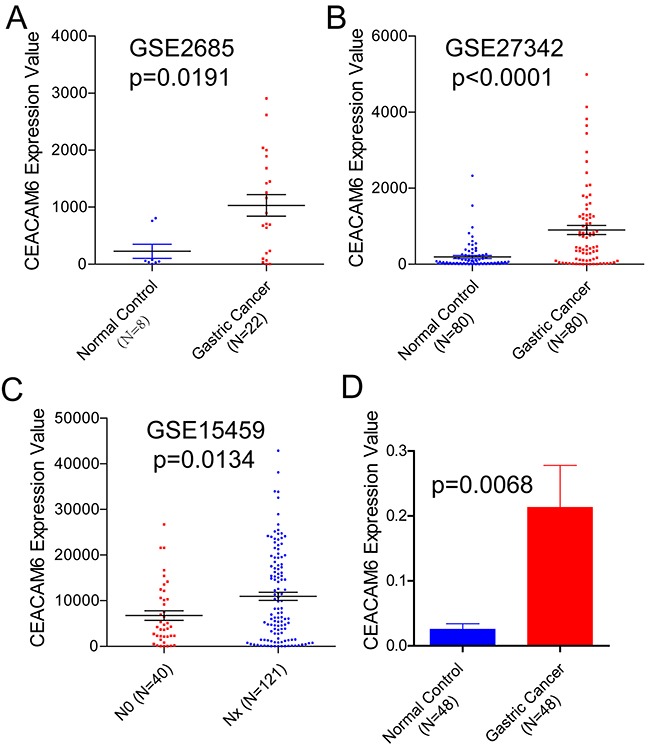
CEACAM6 mRNA expression levels in three different cohort from GEO database **(A)** CEACAM6 is over expressed in 22 gastric cancer tissues compared to 8 noncancerous gastric tissues in GSE2685 dataset (unpaired t-test, p=0.0191, 228.2±122 vs 1032±188). **(B)** CEACAM6 is over expressed in 80 gastric cancer tissues compared to 80 noncancerous gastric tissues in GSE27342 dataset (p<0.0001, 192.5±39.2 vs 903.7±119.3). **(C)** mRNA expression levels of CEACAM6 is significantly higher in lymph node positive gastric cancer patients (Nx, N=121) than negative patients (N0, N=40) (unpaired t-test, p=0.0134, 6758±1067 vs 10960±899). **(D)** Real time PCR results show that CEACAM6 is significantly higher in cancer tissues compare to paired normal mucosa (paired t-test, p=0.0068).

To explore the relationship between CEACAM6 and gastric cancer, three datasets of gene expression profiles (accession: GSE2685, GSE27342 and GSE15459) were downloaded from GEO database. Briefly, GSE2685 has 22 gastric cancer specimens and 8 normal noncancerous controls, GSE27342 contains 80 gastric cancer specimens and 80 adjacent controls, and GSE15459 has 40 lymph node metastasis free (N0) gastric cancer specimens and 121 lymph node metastasis positive (Nx) gastric cancer specimens. As is shown in Figure 1, CEACAM6 is overexpressed in gastric cancer samples compared to noncancerous gastric mucosa controls in datasets GSE2685 and GSE27342 with p=0.0191 and p<0.0001, respectively (Left, Middle). Interesting, CEACAM6 is also positively correlated with lymph node metastasis of gastric cancer (p=0.0134, Figure 1, Right). To sum up, data mining results show that CEACAM6 is over expressed in gastric cancer and is correlated with cancer metastasis.

### IHC validation of the correlation between CEACAM6 and GC

**Figure 2:**
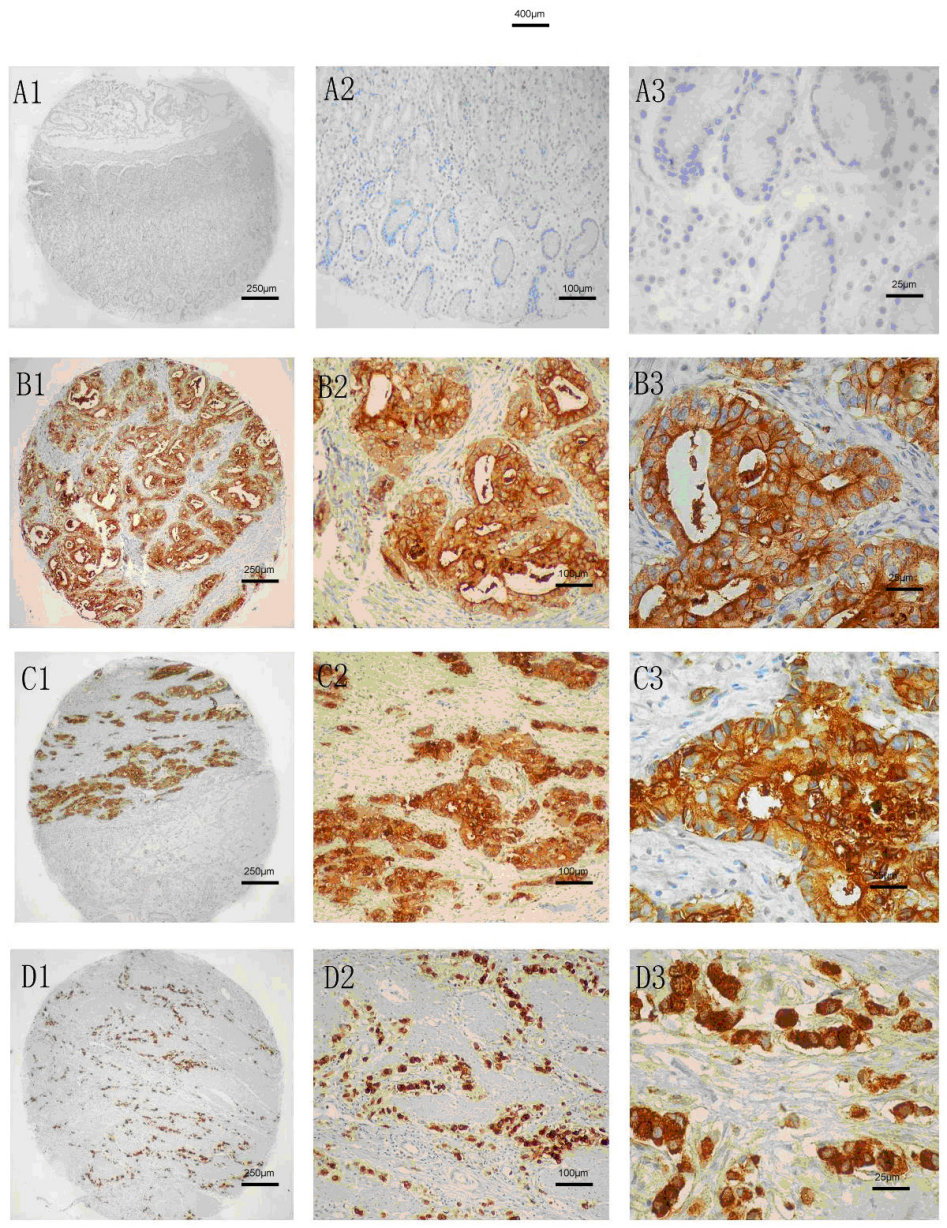
CEACAM6 staining analysis in normal and gastric cancer tissues **(A)** LowCEACAM6 protein expression in normal gastric tissue. **(B)** CEACAM6 protein expressed in tubular adenocarcinoma tissue. **(C)** CEACAM6 protein expressed in poor differentiated GC tissue. **(D)** CEACAM6 protein expressed in signet-ring cell carcinoma tissue. 1: 40×; 2: 100 ×; 3: 400×, Methods: SP.

**Figure 3:**
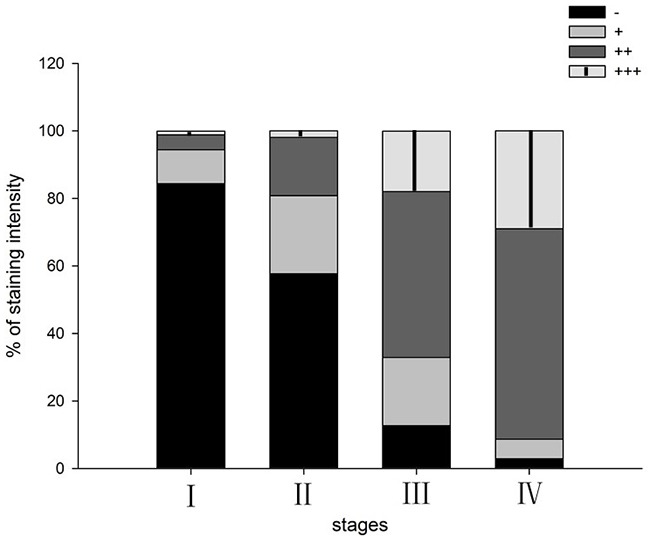
CEACAM6 positive expression distribution in gastric cancer at different TNM stages

Immunohistochemistry results revealed that the CEACAM6 protein level was low or negative in the normal mucosa (Figure 2A). The proportion of CEACAM6 positive specimens was 51.4% (224/436) in the gastric carcinoma specimens and yellow-brown CEACAM6 granules were observed mainly in the cytoplasm (Figure 2B-2D). The proportion of positive CEACAM6 statistically correlated with tumor size, Lauren's classification, vascular invasion, lymph node metastasis, distant metastasis, and TNM stage (Table 1). The ratio of CEACAM6 positivity was 84.4% (120/180) in patients with tumor size ≥5 cm, which was higher than the ratio in patients with tumor size <5 cm (32.8%, 84/256; *p* = 0.000). The frequency of CEACAM6 positive in patients with diffused type GC (78.9%, 168/213) was significantly higher than that with intestinal type (16.1%, 36/223, *p* = 0.003). The rate of samples positive for CEACAM6 was 62.9% (78/124) in gastric carcinoma specimens with poorly differentiated tumors, which was higher than that with well to moderately differentiated tumors (35.3%, 24/68, *p* < 0.001). CEACAM6 was detected in 65.9% (178/270) of GC specimens with lymph node metastasis, which was higher compared with those without lymph node metastasis (15.7%, 26/166, *p*=0.000). The percentage of samples positive for CEACAM6 was 90.2% (55/61) in specimens with distant metastasis in contrast to those without distant metastasis (39.7%, 149/375, *p* < 0.001). CEACAM6 was also significantly detected in 5.6% (5/90) in TNM stage I, 19.2% (20/104) in TNM stage II, 67.1% (116/173) in TNM stage III, and 91.3% (63/69) in TNM stage IV (*p* < 0.001; Figure 3).

**Table 1:** Correlation between CEACAM6 protein expression and clinicopathological features of GC

Parameters	cases	CEACAM6 expression		χ^2^	P
		Negative (%)	Positive (%)		
Tumor diameter				48.652	< .001
< 5	256	172 (67.2%)	84 (32.8%)		
> =5	180	60 (15.6%)	120 (84.4%)		
Lauren classification				172.187	< .001
Diffuse type	223	187 (83.9%)	36 (16.1%)		
Intestinal type	213	45 (21.1%)	168 (78.9%)		
TNM stage				176.634	< .001
I	90	85 (94.4%)	5 (5.6%)		
II	104	84 (80.8%)	20 (19.2%)		
III	173	57 (32.9%)	116 (67.1%)		
IV	69	6(8.7%)	63 (91.3%)		
Vascular invasion				121.548	< .001
No	183	106 (57.9%)	77 (42.1%)		
Yes	253	23 (9.1%)	230 (90.9%)		
Lymph node metastasis				104.314	< .001
No	166	140 (84.3%)	26 (15.7%)		
Yes	270	92 (34.1%)	178 (65.9%)		
Distant metastasis				53.594	< .001
No	375	226 (60.3%)	149 (39.7%)		
Yes	61	6 (9.8%)	55 (90.2%)		

### CEACAM6 is correlated with recurrence and survival of patients with GC

**Figure 4:**
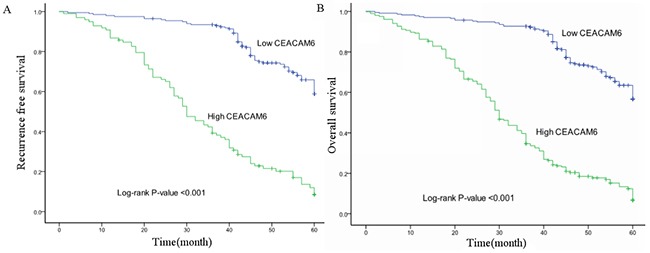
Kaplan-Meier survival curve analysis of patients with gastric cancer positive and negative for CEACAM6 protein expression (log-rank test) Cumulative recurrence-free survival differences **(A)** or cumulative overall survival differences **(B)** between patients with high and low levels of CEACAM6 protein expression. The *p*-value was obtained using the log-rank test of the difference.

Kaplan-Meier survival curves indicated that patients with high CEACAM6 expression exhibited a greater probability of attaining shorter recurrence-free survival (*p* < 0.001, Figure 4A) and overall survival (*p* < 0.001, Figure 4B) than those with low CEACAM6 expression. This result suggests the possible association of CEACAM6 overexpression with poor clinical prognosis. The mean recurrence-free survival time in patients positive for CEACAM6 was 33.16 ± 1.72 months, which is significantly lower than that in patients negative for CEACAM6 (53.68 ± 0.82 months, *p* < 0.001). The mean overall survival time in patients positive for CEACAM6 was 31.98 ± 1.18 months, which is significantly lower than that in patients negative for CEACAM6 (53.17 ± 0.81 months, *p* < 0.001). In addition to CEACAM6 expression, tumor stage was also a significant predictor of recurrence and overall survival (*p* < 0.001). These results were supported further in a multivariate Cox regression analysis, suggesting that the CEACAM6 protein may be involved in the invasion and progression of human GC. Cox multivariate analysis showed that survival was independently correlated with CEACAM6 expression (χ^2^ = 7.740, *p* = 0.005; Table 2).

**Table 2:** Cox multivariate analysis of factors associated with GC survival

Factor	B-value	SE-value	Wald	*p-value*
Tumor diameter	0.13	0.137	0.905	0.341
Lauren classification	0.336	0.211	2.751	0.115
Histology classification	-0.012	0.089	0.017	0.896
Differentiation	0.012	0.135	0.008	0.93
Lymph node metastasis	0.294	0.284	1.071	0.301
Distant metastasis	0.334	0.21	2.526	0.112
TNM stage	0.501	0.184	7.41	0.006^**^
CEACAM6 protein expression	0.49	0.176	7.74	0.005^**^

^**^p < 0.05

### Hub gene analysis of gene signatures regulated by CEACAM6

**Figure 5:**
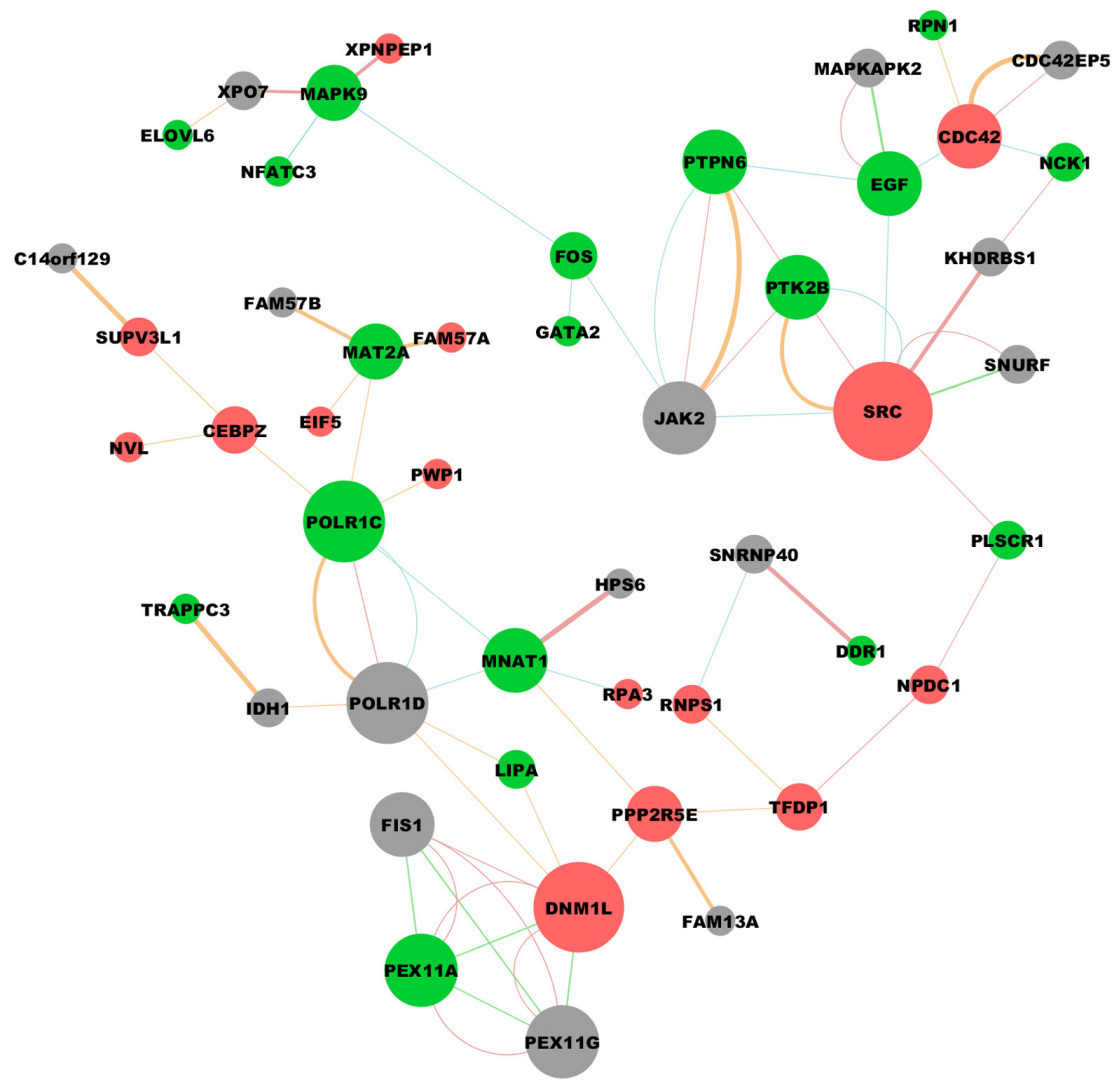
Downstream molecular interaction network regulated by CEACAM6 Red bubble indicates genes up-regulated by CEACAM6 while green indicates down-regulated genes. Grey bubbles means genes that connecting up or down regulated genes which are regulated by CEACAM6. Genes connected with three or more other genes are defined as hub genes which probably play critical functions in the downstream signal network of CEACAM6 (Graph was drawn using Cytoscape software).

Results in previous sections show that CEACAM6 over expressed in gastric cancer samples and positively correlated with gastric cancer progression. To elucidate the potential roles CEACAM6 play in gastric cancer development and progression, we obtained downstream gene signatures of CEACAM6 from LINCs database. Then, gene interaction network was constructed and analyzed through GENEMANIA plugin in Cytoscape environment. As shown in Figure 5, red bubble indicates genes up-regulated by CEACAM6 while green indicates down-regulated genes. Grey bubbles means genes that connecting up or down regulated genes which are regulated by CEACAM6. The size of those bubbles is correlated with number of edges connecting each gene. Genes connected with three or more other genes are defined as hub genes. Full list of hub genes and their molecular functions are shown in Table 3 (degree means number of edges connecting each gene). These hub genes such as SRC (Proto-Oncogene C-Src), DNM1L (Dynamin 1-Like) and POLR1C (polymerase (RNA) I polypeptide C) are critical downstream effectors of CEACAM6 in gastric cancer progression.

**Table 3:** Hub genes of downstream signaling network regulated by CEACAM6

Gene symbol	Function	Degree	Regulation
SRC	regulation of T cell activation|apoptotic signaling pathway	9	Up
DNM1L	regulation of apoptotic signaling pathway	8	Up
POLR1C	transcription initiation from RNA polymerase I promoter	7	Down
PEX11A	peroxisome organization	6	Down
EGF	ERK1 and ERK2 cascade|growth factor receptor binding	5	Down
MNAT1	G1/S transition of mitotic cell cycle	5	Down
PTPN6	regulation of ERK1 and ERK2 cascade	5	Down
PTK2B	stress-activated MAPK cascade	5	Down
CDC42	regulation of T cell activation	5	Up
MAPK9	positive regulation of immune response|MAPK cascade	4	Down
MAT2A	methionine adenosyltransferase activity	4	Down
PPP2R5E	Wnt Signaling Pathway	4	Up
TFDP1	G1/S transition of mitotic cell cycle	3	Up
FOS	positive regulation of immune response|MAPK cascade	3	Down
CEBPZ	Direct p53 effectors	3	Up

### Construction of CEACAM6-drug network

Since CEACAM6 has critical implications in gastric cancer development and metastasis, we searched LINCs database for available drugs that could down regulated CEACAM6 expression. Searching results are displayed as a network in Supplementary Figure 1, which shows that drugs such as Amsacrine, AG-879 (HER2 inhibitor), Wortmannin (PI3K inhibitor) and Tamoxifen could repress the expression of CEACAM6. These drugs have the potential to be used for managing gastric patients with CEACAM6 over expression.

## DISCUSSION

Early detection, accurate staging, and constant disease monitoring remain the prerequisites of effective treatment against GC. Novel GC detection and monitoring methods are urgently needed because current biomarkers, such as CA19-9, lack enough sensitivity and specificity. The carcinoembryonic antigen (CEA) family is overexpressed in various epithelial derived cancer types, and their deregulation could promote metastasis in animal models [21, 25]. Currently, CEACAM1, CEACAM5, and CEACAM6 are considered as valid prognostic markers and promising therapeutic targets in melanoma, lung, colorectal, and pancreatic cancers [26].

CEACAM6 belongs to of glycosylphosphatidy-linositol-linked (GPI-linked) Ig superfamily and mainly expressed in neutrophils and some epithelial cells [21]. Studies demonstrated that CEACAM6 is overexpressed in several human malignancies, including colorectal adenomas and carcinomas, gastric carcinomas, and pancreatic carcinomas etc.[17, 27–29]. In pancreatic cancer cells, CEACAM6 overexpression was associated with anoikis resistance and tumor metastasis *in vitro* and *in vivo* [30]. Researchers also tested a single domain antibody targeting CEACAM6 that could be an ideal candidate for treating pancreatic cancer with CEACAM6 overexpression [31]. In colon cancer, CEACAM6 overexpression prevents colonocyte differentiation and promotes oncogenesis in nude mice [32]. CEACAM6 is also overexpressed in breast cancer, and its expression in atypical ductal hyperplasia may be a biomarker for the invasiveness of breast cancer [33, 34].

Inhibition the expression of CEACAM6 by antibodies or RNAi can repress tumor cell growth, adhesion, invasion and metastasis, resulting in improved survival of mice carrying tumors [29, 33]. Zhang et al. demonstrated that CEACAM6 overexpression could promote migration and invasion of GC cells *in vitro* and their also showed that CEACAM6 expression was correlated with metastases by performing IHC on 101 pair-matched GC patient samples [35]. A study from the same group showed that CEACAM6 could promote gastric cancer invasion and metastasis by inducing Epithelial-mesenchymal transition [36]. Another research group also demonstrated that CEACAM6 was associated with the tumorigenesis and lymph node metastasis [37]. Recently, a study by Roy and co-authors indicated that CEACAM6 could be upregulated by Helicobacter pylori CagA and was a biomarker for early gastric cancer [38]. However, the sample size of these studies are relatively small and validation with a large cohort of GC are needed. In this study, we show that the mRNA and protein levels of CEACAM6 are significantly up regulated in GC. The proportion of CEACAM6 positive patients is 51.4% (224/436) in the gastric carcinoma specimens. The protein level of CEACAM6 is significantly correlated with tumor size, Lauren's classification, invasion depth, lymph node metastasis, distant metastasis, and TNM stage. We also find that the median survival time and the five-year survival rate in CEACAM6 (+) patients is significantly shorter than CEACAM6 (-) patients. These results suggest that CEACAM6 may be used as an predictive biomarker of invasion, metastasis, and poor prognosis of GC.

Moreover, the present study provided new insights into the multiplicity and diversity of CEACAM6 expression and their potential functions in tumor development and progression. The median survival (recurrence and overall) time and five-year survival rate in CEACAM6 positive patients were significantly lower than CEACAM6 negative patients. Multivariate cox analysis results further showed that overall survival was independently correlated with CEACAM6 expression.

Network analysis of downstream gene signatures of CEACAM6 discovered several hub genes which may play critical roles in mediating tumor proliferation and metastasis. Of those hub genes, DNM1L is a member of the dynamin superfamily of GTPases, and is involved in developmentally regulated apoptosis and programmed necrosis [39]. Dysfunction of this gene is implicated in several neurological disorders, including Alzheimer's disease [40]. Recent studies show that DNM1L could repress apoptosis of cancer cells and promote migration and invasion in breast cancer [41–43]. However, the function of DNM1L in gastric cancer has never been explored. This gene can be an important downstream effector of CEACAM6 and mediating gastric cancer progression. Furthermore, we found several available drugs that could inhibit the expression of CEACAM6, which in turn may lead to the repression of gastric cancer progression. For instance, Tamoxifen is the most widely used drug in breast cancer management with barely no side effects [44, 45]. The application of Tamoxifen in combination with traditional chemotherapeutics in the treatment for CEACAM6 over expression gastric cancer patients may generate positive benefits.

In summary, CEACAM6 overexpression is a common characteristic in GC and is positively associated with metastasis and poor prognosis of GC. Currently available drugs such as Tamoxifen and AG-879 could be used for managing patients with CEACAM6 over expression. Nevertheless, further wet lab experiments and appropriately designed clinical trials are still needed before the application of CEACAM6 as a biomarker and therapeutic target.

## MATERIALS AND METHODS

### Ethics statement

All of the study protocols in this study were approved by the Institutional Ethics Committee of the Zhejiang Provincial People's Hospital, Hangzhou, China. Written informed consent was obtained from the next of kin, caretakers, or guardians on behalf of minors/child participants. Permission for using the information in the medical records of the patients for research purposes was obtained from the Zhejiang Provincial People's Hospital. The Institutional Ethics Committee of the Zhejiang Provincial People's Hospital also ensured that relevant ethical issues in this study were considered.

### Datasets

Gene expression profiles of gastric cancer samples and normal controls were obtained from GEO (Gene Expression Omnibus: http://www.ncbi.nlm.nih.gov/geo/) database [46]. Accession number: GSE2685 (22 gastric cancer specimens vs 8 normal noncancerous controls), GSE27342 (80 gastric cancer specimens vs 80 normal noncancerous controls) [47] and GSE15459 (40 lymph node metastasis free gastric cancer specimens vs 121 lymph node metastasis positive gastric cancer specimens) [48]. Gene signatures regulated by CEACAM6 and Drugs targeting CEACAM6 were obtained from LINCs database [49].

### Tumor samples

The paraffin specimens of tumors were obtained from 436 patients with GC (17 to 91 years old, no radiotherapy or chemotherapy treatment before operation) who underwent curative gastrectomy between 1998 and 2004 at the Department of General Surgery (Zhejiang Provincial People's Hospital, Hangzhou, China). All of the cases were classified according to the WHO Pathological Classification of Tumors. Patients were follow up for over five years after operation. Detailed information about these samples are described previously [50].

In addition, 48 fresh frozen cancer tissues and surrounding normal gastric mucosa were obtained from patients with GC at Zhejiang Provincial People's Hospital from January 2007 to December 2008 and stored at -80°C until use.

### Immunohistochemical staining and evaluation

Immunohistochemical staining of CEACAM6 was performed with rat anti-human CEACAM6 (1:150, Abcam, GBR) using protocols described in detail previously [51]. Immunohistochemically stained sections were reviewed and evaluated by two independent pathologists. All of the slides were observed under a Nikon light microscope (Nikon Corporation, Tokyo, Japan), and representative photographs were captured.

### Real-time PCR

Real-time PCR was conducted according to the user's manual of the PCR kit. In brief, total RNA was extracted from the fresh cancer tissues and the surrounding normal gastric mucosa by using Trizol reagent (Invitrogen, USA). To generate cDNA, 1μg total RNA was reverse-transcribed using PrimeScript First Strand cDNA Synthesis kit (Takara, DRR047A, Japan) in a total reaction volume of 20 μl according to the manufacturer's instructions. Real-time PCR was performed using the MX3000P Real-time RCR Detection System (Stratagene, USA) by using gene-specific primers with SYBR Premix ExTaq kit (Takara, Japan). The forward and reverse primer of CEACAM6 were 5’-GGGTATCGCTGAGACTAAGTTGTA-3’ and 5’-CCTTAGGCAAGATACAAACCAAC-3’, respectively. Human glyceraldehyde-3-phosphate dehydro genase (GAPDH) was used as an internal control for real-time PCR. The primer sequence of GAPDH was 5’-cgattggatggtttagtgagg-3’ (forward) and 5’-agttcgaccgt cttctcagc-3’ (reverse; Invitrogen).

After 30 s of initial denaturation at 95 °C, 40 cycles of amplification were performed at 95 °C for 5 s, 55 °C 20 s, and 70 °C for 20 s. At the end of the PCR cycles, melting curve analyses were performed. The intensity of the dye fluorescence was determined, and the expression levels of these mRNAs in relation to GAPDH were calculated using 2^-ΔCt^ method.

### Statistical and bioinformatics analyses

Data were statistically analyzed using SPSS software (Version 21.0). Results were presented as mean ± standard deviation (SD); Paired sample *t*-test or one-way ANOVA was employed as appropriate. Categorical variables were presented as percentages and analyzed by Fisher's exact test. Survival curves were plotted using Kaplan-Meier method and compared hazards method.

Gene expression profiles were processed through R software [52]. Scatter plots and related statistical analyses were made through Graphpad prism (version 5.0, GraphPad Software, Inc). GENEMANIA plugin [53] in Cytoscape environment (version 3.1.1) [54] was employed to build the interaction network of CEACAM6 regulated gene signatures. Hub gene was defined as genes that interacts with 3 or more other genes. CEACAM6-drug network was also constructed using Cytoscape. All *p*-values resulted from the use of two-sided statistical tests, and differences were considered significant at *p* < 0.05.

## SUPPLEMENTARY MATERIALS FIGURE


