# Supplementary material for: CEACAM6 is a prognostic biomarker and potential therapeutic target for gastric carcinoma
Source: Oncotarget. 2017 Jul 20;8(48):83673–83. doi: 10.18632/oncotarget.19415 (PMC5663545; doi:10.18632/oncotarget.19415)
Supplement: Supplementary file 1 [file oncotarget-08-83673-s001.pdf]

CEACAM6 is a prognostic biomarker and potential therapeutic target for gastric carcinoma

SUPPLEMENTARY MATERIALS

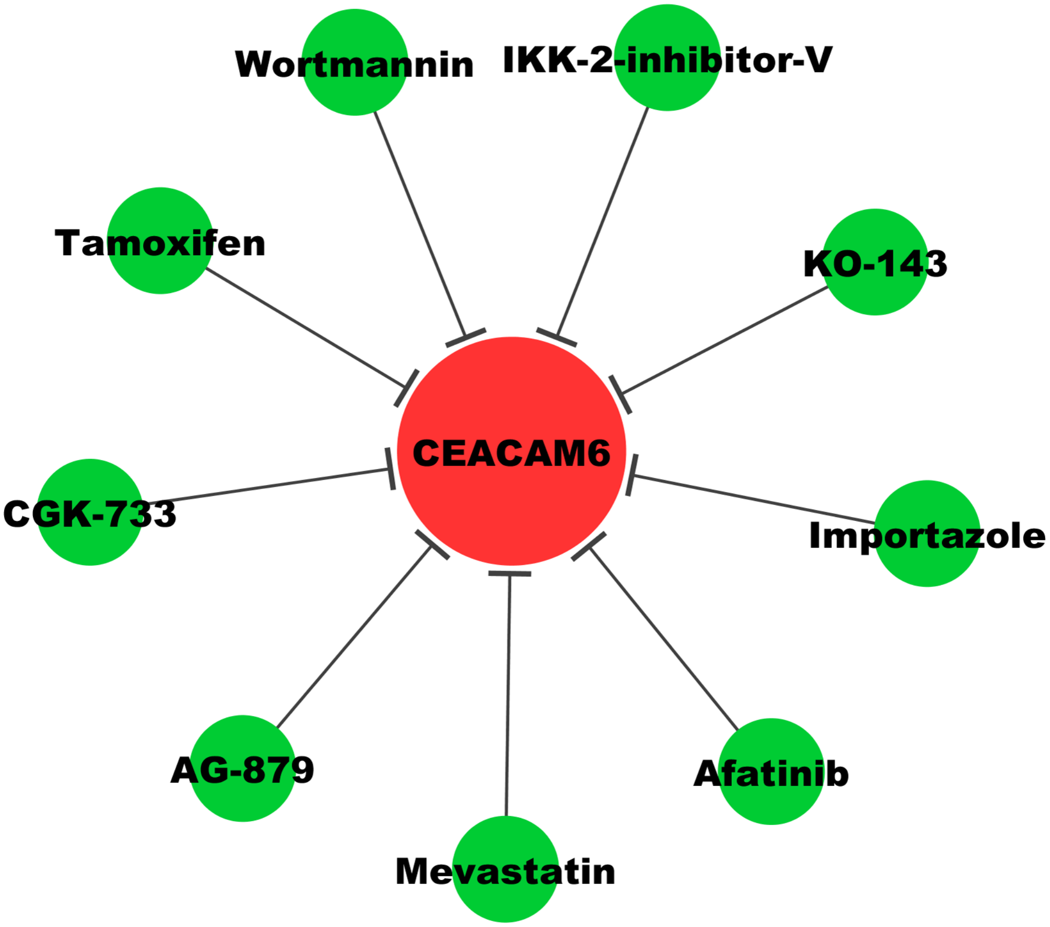

Supplementary Figure 1: Drugs that could down-regulate CEACAM6 expression. As is shown in this network, Drugs such as Amsacrine, AG-879 (HER2 inhibitor), Wortmannin (PI3K inhibitor) and Tamoxifen could repress the expression of CEACAM6.
